# Supplementary material for: Factors associated with depression among heart failure patients at selected public hospitals in Addis Ababa, Ethiopia: A cross sectional study
Source: PLOS Glob Public Health. 2022 Aug 4;2(8):e0000853. doi: 10.1371/journal.pgph.0000853 (PMC10021377; doi:10.1371/journal.pgph.0000853)
Supplement: Attachment — Submitted filename: 1. Response to editorial comments.docx [file pgph.0000853.s001.docx]

**Date:** 15/01/2022

**From:** Corresponding author

**To**: "PLOS Global Public Health" globalpubhealth@plos.org

**Ref:** PLOS Global Public Health: Edits requested on your submission PGPH-D-22-00002 - [EMID:2c23ebf88a2df1ea]

**Subject**: Response to Edits requested on submission PGPH-D-22-00002

**Title of Manuscript:** Prevalence of depression and associated factors among heart failure patients at cardiac follow-up clinics in a government teaching hospital at Addis Ababa, Ethiopia; A cross sectional study

Dear Editorial office of PLOS Global Public Health

Initially, on behalf of the authors I would like to take this opportunity to thank to the editorial office of PLOS Global Public Health for your valuable and constructive comments on the grammar check and spelling error to the entire document review. It is obviously clear that PLOS one journal plays a vital part in publishing original article for academic community across the world, that is why we prefer it. In addition to this we proud of you in timely response, providing valid and relevant comments during review process, journals indexed in known databases (PubMed, Scopus, Web of Science), high impact factor and possession of clear manuscript submission guideline are some of the good aspects of PLOS one Journal. We look forward to work with you and keep it up.

Hopefully all of the required comments are corrected point by point in line with the given direction and uploaded to the submission system.

Once again presenting thank you honestly for your work and wishing all the best for future endeavors.

Sincerely,

The authors.
