# Supplementary material for: Factors associated with depression among heart failure patients at selected public hospitals in Addis Ababa, Ethiopia: A cross sectional study
Source: PLOS Glob Public Health. 2022 Aug 4;2(8):e0000853. doi: 10.1371/journal.pgph.0000853 (PMC10021377; doi:10.1371/journal.pgph.0000853)
Supplement: Attachment — Submitted filename: 4. A rebuttal Letter .docx [file pgph.0000853.s002.docx]

**Date: 05**/03/2022

**From:** Corresponding authors

**To**: "PLOS Global Public Health" globalpubhealth@plos.org

**Ref:** PLOS Global Public Health: Revised Report Submission PGPH-D-22-00002 - [EMID:2c23ebf88a2df1ea]

**Subject**: A rebuttal letter that responds to each point raised by editor and reviewer submission PGPH-D-22-00002

**Title of Manuscript:** Prevalence of depression and associated factors among heart failure patients at cardiac follow-up clinics in a government teaching hospital at Addis Ababa, Ethiopia; A cross sectional study

Dear Editorial office of PLOS Global Public Health

Initially, we would like to take this opportunity to thank to the editorial office of PLOS Global Public Health **journal** editorial office and reviewers for your valuable comments that raised in the given manuscript. All the given comments are amended based on the given directions.

Both marked with high light document and unmarked one uploaded to the submission system.

Wishing you all the best for future endeavors.

Sincerely,

The authors.
