# Supplementary material for: Factors associated with depression among heart failure patients at selected public hospitals in Addis Ababa, Ethiopia: A cross sectional study
Source: PLOS Glob Public Health. 2022 Aug 4;2(8):e0000853. doi: 10.1371/journal.pgph.0000853 (PMC10021377; doi:10.1371/journal.pgph.0000853)
Supplement: Attachment — Submitted filename: Letter in response to reviewer comments.pdf [file pgph.0000853.s003.pdf]

Date: 17 June 2022

From: Corresponding authors

To: "PLOS Global Public Health" globalpubhealth@plos.org

Ref: PLOS Global Public Health:

Revised Report Submission PGPH-D-22-00002 -[EMID:2c23ebf88a2df1ea]

Subject: Letter of response to reviewer

Title of Manuscript: Prevalence of depression and associated factors among heart failure patients at cardiac follow-up clinics in a government teaching hospital at Addis Ababa, Ethiopia; A cross sectional study.

Dear Editorial office of PLOS Global Public Health

thank you to all editorial office staffs and reviewers for your incredible support in reviewing and academic suggestion on the identified gaps.

For your further review and approval here submitted final document in the manuscript submission system.

Sincerely,

The authors.
